# Dynamic model assuming mutually inhibitory biomarkers of frailty suggests bistability with contrasting mobility phenotypes

**DOI:** 10.3389/fnetp.2023.1079070

**Published:** 2023-05-04

**Authors:** Nathan Schaumburger, Joel Pally, Ion I. Moraru, Jatupol Kositsawat, George A. Kuchel, Michael L. Blinov

**Affiliations:** ^1^ Department of Ecology and Evolutionary Biology, University of Connecticut, Storrs, CT, United States; ^2^ Center for Cell Analysis and Modeling, UConn Health, Farmington, CT, United States; ^3^ UConn Center on Aging, UConn Health, Farmington, CT, United States

**Keywords:** mathematical model, dynamical model, aging, IL-6, IGF-1, frailty, bistabiity, phenotype

## Abstract

Bistability is a fundamental biological phenomenon associated with “switch-like” behavior reflecting the capacity of a system to exist in either of two stable states. It plays a role in gene regulation, cell fate switch, signal transduction and cell oscillation, with relevance for cognition, hearing, vision, sleep, gait and voiding. Here we consider a potential role for bistability in the existence of specific frailty states or phenotypes as part of disablement pathways. We use mathematical modeling with two frailty biomarkers (insulin growth factor-1, IGF-1 and interleukin-6, IL-6), which mutually inhibit each other. In our model, we demonstrate that small variations around critical IGF-1 or IL-6 blood levels lead to strikingly different mobility outcomes. We employ deterministic modeling of mobility outcomes, calculating the average trends in population health. Our model predicts the bistability of clinical outcomes: the deterministically-computed likelihood of an individual remaining mobile, becoming less mobile, or dying over time either increases to almost 100% or decreases to almost zero. Contrary to statistical models that attempt to estimate the likelihood of final outcomes based on probabilities and correlations, our model predicts functional outcomes over time based on specific hypothesized molecular mechanisms. Instead of estimating probabilities based on stochastic distributions and arbitrary priors, we deterministically simulate model outcomes over a wide range of physiological parameter values within experimentally derived boundaries. Our study is “a proof of principle” as it is based on a major assumption about mutual inhibition of pathways that is oversimplified. However, by making such an assumption, interesting effects can be described qualitatively. As our understanding of molecular mechanisms involved in aging deepens, we believe that such modeling will not only lead to more accurate predictions, but also help move the field from using mostly studies of associations to mechanistically guided approaches.

## 1 Introduction

### 1.1 Bistability as a hallmark of biological systems

The rate at which individuals age and become disabled varies (Guralnik et al., 1996). Aging trajectories are often nonlinear, with growing evidence to indicate that specific states of increased vulnerability or frailty with distinct phenotypes may exist in selected older adults (Fried et al., 2001; Bouchonville and Villareal, 2013). Aging (Lopez-Otin et al., 2013), chronic diseases of aging (Lopez-Otin et al., 2013) and geriatric syndromes (Inouye et al., 2007) are all highly multifactorial, requiring the development of computational and modeling approaches to help define the manner in which different biomarkers may interact with each other and influence trajectories of aging and the emergence of specific clinical states.

Both simple and complex systems often exist in either of two stable states (Tyson et al., 2003), which is a fundamental phenomenon of nature called bistability. Bistability has been shown to play a role in many different biological processes ranging from gene regulation (Anderson et al., 2012), cell fate switch (Ferrell et al., 2009), and cell oscillation (Ferrell et al., 2009) to signal transduction (Bagowski and Ferrell, 2001; Brandman and Meyer, 2008). It is often associated with the presence of feedback regulation involving opposing pathways (Ferrell, 2008). Moreover, it has also been implicated in a number of physiological responses which represent key determinants of function and independence in late life. These include working memory (Durstewitz and Seamans, 2006), hearing (Kondo and Kochiyama, 2017), vision (Kondo and Kochiyama, 2017), sleep (Selbach et al., 2010), gait (Engbers et al., 2013), and voiding (Stibitz, 1967; Hosein and Griffiths, 1990).

Dynamic modeling has been used by systems biologists to develop a quantitative understanding of complex intracellular signaling networks (Moraru et al., 2008). To investigate whether bistability plays a role in the emergence of different states of mobility disability during aging, we sought to build a predictive dynamic model involving selected biomarker interactions that were reflective of clinically relevant outcomes, and that were also associated with properties likely to be conducive to the emergence of bistability. To that end, we identified biomarkers previously shown to demonstrate evidence of significant interactions in their associations with mobility performance, a critical factor for determining the development and worsening of frailty and functional dependence (Guralnik et al., 1996). For our deterministic modeling, we have defined the state of normal mobility performance as falling within published age-adjusted population norms for standardized measures of mobility performance, and to that end we have used a walking speed of less than 0.4 m/s to define limited mobility performance (Cappola et al., 2003). We searched for biomarkers that were reflective of mutually-inhibitory pathways since their existence can create conditions leading to the emergence of bistability (Ferrell, 2002; Brandman and Meyer, 2008), where a system behaves like a toggle switch between two different states.

Thus, biomarker pairs involving opposing associations with mobility disability and mutually inhibitory pathways were of particular interest. Of those, high peripheral interleukin-6 (IL-6) levels, and low insulin growth factor 1 (IGF-1) levels indicated evidence of mutually inhibiting associations with mobility disability (Cappola et al., 2003).

### 1.2 IL-6 and IGF-1: contrasting associations with mobility disability.

Elevated IL-6 levels in peripheral blood represent a validated predictor of declining mobility performance in older adults (Cohen et al., 1997). IL-6 is a pleiotropic cytokine, which in addition to T-cells and macrophages is also produced by many non-immune cells (Scheller et al., 2011; Kistner et al., 2022). It is easily measured in the peripheral blood where its levels are indicative of overall production from several tissues including fat, liver, and muscle (Scheller et al., 2011). Nevertheless, in the local microenvironment, IL-6 levels are carefully regulated, exerting either pro- or anti-inflammatory effects depending on the acuity of change and the tissue or intervention being studied (Scheller et al., 2011). Its effects on skeletal muscle are complex (Scheller et al., 2011; Kistner et al., 2022), yet most germane to our work are studies showing that IL-6 promotes both muscle catabolism (Bakker and Jaspers, 2015) and insulin resistance (Pedersen and Edward, 2009), with chronic IL-6 administration inducing skeletal muscle atrophy (Haddad et al., 2005). At the same time, acute increases in IL-6 contribute to the induction of skeletal muscle stem cell responses after exercise (Toth et al., 2011), while in the absence of all IL-6 the recovery from disuse atrophy (Washington et al., 2011) and overload-induced hypertrophy (Serrano et al., 2008) are both decreased.

IL-6 and IGF-1 demonstrate opposing associations and roles in mobility performance. Higher IL-6 levels have been linked to declines in mobility performance, while higher IGF-1 levels are associated with maintained mobility performance. (Cappola et al., 2001). IGF-1 is a hormone that is produced mostly in the liver with synthesis regulated by growth hormone (Bakker and Jaspers, 2015). In addition to its systemic endocrine effects, IGF-1 is also produced locally exerting paracrine or autocrine effects in many tissues (Bakker and Jaspers, 2015). In skeletal muscle, IGF-1 stimulates muscle fiber hypertrophy via increased protein synthesis (Semsarian et al., 1999) and decreased protein degradation (Glass, 2010). Mechanical stretching and muscle contraction induce the production of IGF-1 isoforms called MGF E and IGF-1 Ea (Heinemeier et al., 2007), with the former stimulating muscle stem cell proliferation (Mills et al., 2007) and capacity for regeneration (Bakker and Jaspers, 2015), while the latter promotes muscle stem cell differentiation into myotubes (Mills et al., 2007).

### 1.3 IL-6 and IGF-1: synergistic and mutually-inhibitory biomarkers for mobility disability

Epidemiologists view synergy as an example of effect modification or positive interaction in which joint effects exceed the sum of separate effects (Rothman, 1995). Cappola et al. (2003) sought to evaluate the combined effects of different IGF-1 and IL-6 levels on the risk of mobility disability in older women They evaluated both the independent and combined effects of levels of these biomarkers in a cohort of 718 community-dwelling women 65 years and older. When stratifying subjects according to IL-6 and IGF-1 levels, women who were in both the highest quartile for IL-6 and the lowest quartile for IGF-1 were at far greater risk for disability when compared to their counterparts who only had either one of these two biomarkers.

It is noteworthy that IL-6 and IGF-1 appear to be inversely correlated with each other. For example, transgenic mice overexpressing IL-6 in all cells demonstrate stunted growth which is mediated through decreased IGF-1 levels and signaling (De Benedetti et al., 1997). In contrast, low IGF-1 levels are associated with chronic inflammation, while growth hormone replacement reduces both peripheral inflammatory marker levels (Sesmilo et al., 2000) and monocyte activation (Serri et al., 1999).

These two biological examples demonstrated evidence for this inverse correlation via mutual inhibition (Bakker and Jaspers, 2015). For example, systemic elevations in IL-6 may lower IGF-1 levels at least in part through increased clearance (De et al., 2001). In muscle, cross-talk between IL-6 and IGF-1 associated pathways may also occur through activation of SOCS-3 (Al-Shanti et al., 2008; Al-Shanti and Stewart, 2012), a cytokine-inducible negative regulator of cytokine signaling, with elevated SOCS-3 levels possibly contributing to declines in skeletal muscle stem cell function with aging (McKay et al., 2013). While additional sites of interaction likely remain to be identified, mTOR (mammalian target of rapamycin) provides another potential locus of interaction since IGF-1 stimulates muscle protein synthesis through this pathway, while IL-6 has the capacity to both upregulate (Bakker and Jaspers, 2015) and downregulate mTOR activity (White et al., 2012), via Akt and AMPK pathways, respectively (Bakker and Jaspers, 2015).

With all the above considerations in mind, we developed a predictive dynamic model based on the concept of mutually inhibiting biomarkers. In our model we assume that mutual inhibition in specific pathways at the cellular levels extrapolates to biomarkers' blood concentrations, which is most likely not a straightforward dependence. Thus, our model can be considered a “demonstrator” on how our knowledge of molecular mechanisms can be potentially translated into a deterministic predictive model.

## 2 Methods

### 2.1 A mathematical model associating IL-6 and IGF-1 with mobility performance.

**FIGURE 1:**
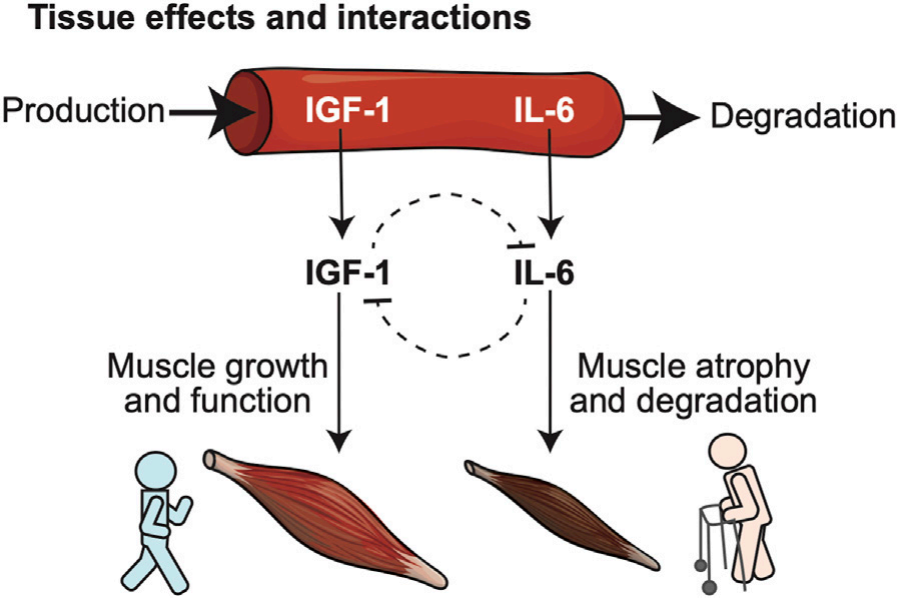
Conceptual model illustrates relationships between biomarkers predictive of frailty, and their opposing biological roles in key physiological determinants of frailty such as muscle health. Two blood-born frailty biomarkers (IGF-1, IL-6) studied in our model were selected for their demonstrated synergism in predicting risk of frailty, disability and death in large cohort studies and their mutually opposing effects at the level of specific target tissues: IGF-1 promotes muscle growth, while IL-6 favors muscle atrophy and degeneration.

We created a mathematical model for blood serum levels of IGF-1 and IL-6 and muscle function (shown at a conceptual level in Figure 1), that recapitulates changes in mobility over time. The model includes 5 time-dependent variables in a hypothetical individual. These included two quantitative biomarkers (blood serum levels of IGF-1 and IL-6), and three variables defining deterministically-computed prevalence scores for an individual to be in one of three specific clinical states: mobile (non-frail), less mobile (having mobility disability), or dead. We define prevalence score as a measure of **individual’s health outcome**, computed as a deterministically-simulated fraction of individuals with the same IGF-1 and IL-6 levels that have a given clinical outcome at certain time.

**FIGURE 2:**
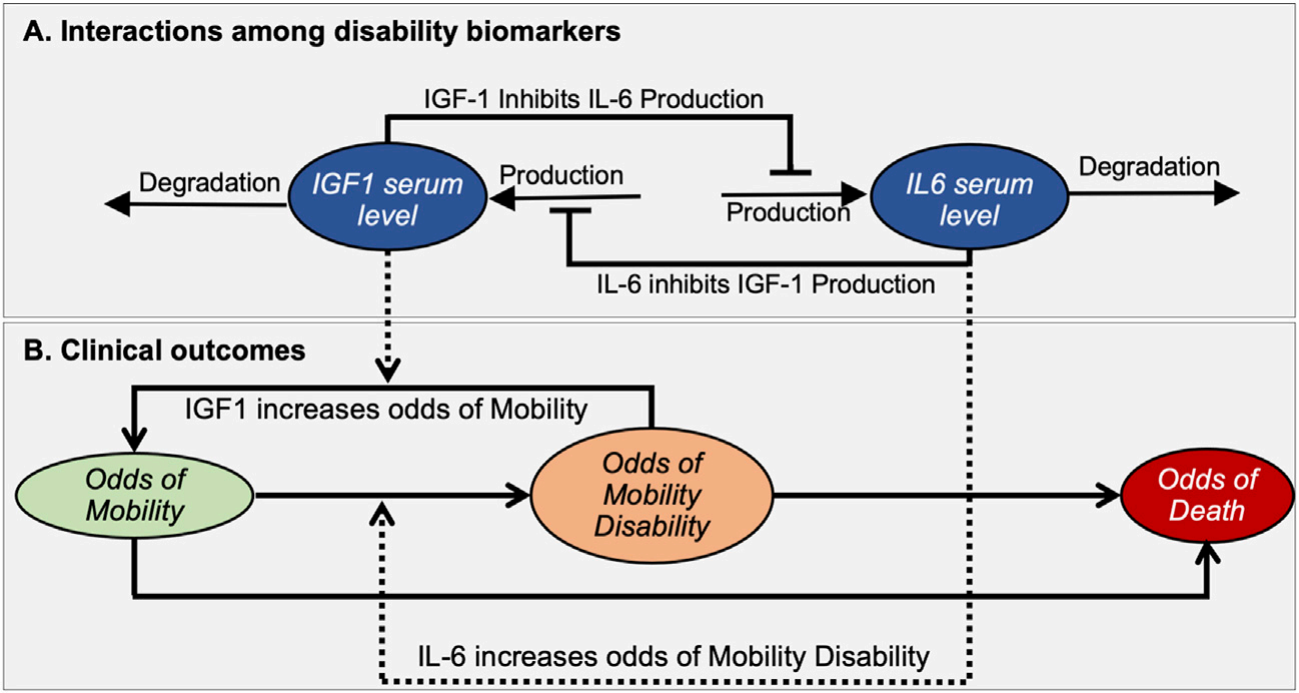
A mathematical model illustrates interactions between disability biomarkers IL-6 and IGF-1 in peripheral blood serum **(A)** and their relationships to the prevalence score of mobility disability **(B)**. The mathematical model describes the dynamic properties of a bistable molecular system driving changes in the prevalence score for an individual to be in Mobile, Mobility Disability or Deceased states. Ovals indicate time-dependent variables. Solid black lines define interactions among biomarkers **(A)** and changing of outcomes **(B)**, while dashed lines indicate how biomarkers affect such transitions. Arrows and bars at the end of lines indicate positive or negative interactions, respectively.

We assume that IGF-1 and IL-6 levels affect each other through several molecular pathways, leading to mutual inhibition of each other. This relationship between IGF-1 and IL-6 is captured in the biomarker interaction module of the model (Figure 2A). It implements the assumption of mutual inhibition in which the production of each factor is inhibited by the other through a negative feedback mechanism. As a result, increased levels of either factor result in decreasing the production rate for the other.

The clinical outcomes module addresses the relationship of these biomarkers to relevant clinical outcomes (Figure 2B). Here we introduced three variables termed “Prevalence score of Mobility” (*M*), “Prevalence score of Mobility Disability” (*MD*), and “Prevalence score of Death” (*D*) that corresponds to the prevalences of an individual to be in a mobile, disabled or dead state respectively. Note that these prevalence scores are not all independent variables, as the sum of all three prevalence scores should be equal to 1. Changes in prevalence scores among these deterministically-determined states are driven by levels of biomarkers. We assume that higher IGF-1 levels favor the maintenance of mobility, while in contrast higher IL-6 levels are associated with increased mobility disability. We also assume that feedback of the resulting clinical state on the molecular interactions is negligible and does not affect the mutual inhibition of biomarkers, thus also not impacting phenotypic outcomes.

We assumed that the remaining life expectancy is the same for the whole population, and that mortality is defined by frailty and not directly affected by the levels of biomarkers. The rationale for not including a direct influence of IGF-1 or IL-6 on mortality in the model is the fact that relationships between these biomarkers and prevalence scores of death from a clinical perspective remain unclear. While lower IGF-1 levels may contribute to higher mortality in humans (Sonntag et al., 2012), higher IGF-1 levels have been linked to many human cancers (Werner and Bruchim, 2012). In contrast, higher IL-6 levels have been linked to increased mortality both in the context of chronic diseases (Lindmark et al., 2001), and infections (Sinha et al., 2021) such as COVID-19.

When describing changes over time of the variables included in this model it is reasonable to assume that they represent continuous quantities. Therefore we can utilize deterministic temporal simulations driven by ordinary differential equations that describe the rate of change of the variables (Resat et al., 2009). Expressed mathematically in our model**,** concentrations of IGF-1 and IL-6 biomarkers at any time *t* are given by functions *IGF1(t*) and *IL6(t)*. The differential equation that governs the rate of change for the variable *IGF1* representing the concentration of IGF-1 in blood serum is:

$$\frac{d\;IGF1}{dt}=\frac{{kp}_{IGF1}}{1+{\left({ks}_{IL6}\;IL6\right)}^{2}}-{kd}_{IGF1}\;IGF1$$

Here *IGF1* is produced with the constant rate *kp*_*IGF1*_, inhibited by the square of the amount of IL-6 scaled by parameter *ks*_*IL6*_ and degraded proportionally to IGF-1’s amount with a rate *kd*_*IGF1*_. We used the Hill-type kinetic law with the Hill coefficient (exponent for inhibitor concentration in the denominator) being 2. In chemical kinetics the Hill coefficient of more than 1 would signify positively cooperative binding—once inhibitor binds, it enables easier binding of the next inhibitor. In our context, it corresponds to an increasing inhibition effect. However, there is no direct analogy, as our model does not describe an exact mechanism of inhibition, but rather an effect of mutually inhibiting pathways.

Similarly, the equation for the variable IL6 is:

$$\frac{d\;IL6}{dt}=\frac{{kp}_{IL6}}{1+{\left({ks}_{IGF1}\;IGF1\right)}^{2}}-{kd}_{IL6}\;IL6$$

The differential equation connecting the prevalence score of mobility and other prevalence scores is based on the linear dependency of the prevalence score, where the rate of change for prevalence score of event A have negative terms proportional to prevalence score of A (when event A is changing to other events) and positive terms proportional to prevalence score of all other events that can be changed to A. The amounts of IGF-1 and Il-6 serve as modifiers for this relationship. Specifically, as a mobile person may become disabled, the rate of change for the prevalence score of Mobility (M) for an individual at each time point has a negative term proportional (with the coefficient *k*_*loss*_) to the level of IL-6 (the higher the level of IL-6, the higher is prevalence score of being disabled) and prevalence of being mobile at this time point. As a disabled person may recover, the rate of change for the prevalence score of Mobility (M) has a positive term proportional (with the coefficient *k*_*gain*_) to the level of IGF-1 and prevalence score of having mobility disability at this time point. Finally, as a mobile person can die, the rate of change for prevalence score of mobility has a negative term proportional to the prevalence score of mobility and is modified by a parameter *k*_*mort.*_ Additionally, mortality increases exponentially with time. This increase is parameterized by *k*_*longevity*_, which sets the maximal survival of the people in the population to be 240 months after the initial measurements of biomarkers, which makes sense for the mean age of 77.6 years old (range is 65–100 years).

$$\frac{d\;M}{dt}=-k_{loss}\;IL6\;M+k_{gain}\;IGF1\;MD-k_{mort}\;e^{k_{longevity}\;t}\;M$$

Similarly, the rate of change of theprevalence score of Mobility Disability (*MD*) of an individual at each time point is increasing proportionally to the amount of IL-6 with a coefficient *k*_*loss*_, decreasing proportionally to the amount of IGF-1 with a coefficient *k*_*gain*_, and decreasing due to mortality. We assume that those with mobility disability have increased mortality prevalence score (compared to the mobile population) defined by a parameter *k*_*extra*_:

$$\frac{d\;MD}{dt}=-k_{gain}\;IGF1\;MD+k_{loss}\;IL6\;M-k_{extra}\;k_{mort}\;e^{k_{longevity}\;t}\;MD$$

The parameters are all unknown and were estimated using published data on IGF-1 and IL-6 levels as validation datasets.

### 2.2 Parameter estimation

The mathematical model described above represents a phenomenological approximation, and therefore parameter values are *a priori* not measurable quantities and are unknown. They were estimated using published data as validation datasets, an approach to mathematical modeling called fitting model parameters to data (Motulsky and Christopoulos, 2004). This was performed using COPASI (Hoops et al., 2006) parameter fitting engines within Virtual Cell (VCell), a problem-solving environment (Moraru et al., 2008). The authors are not aware of any published studies involving a longitudinal design where all relevant variables (biomarkers, mobility metrics, etc.) were monitored simultaneously over an extended period of time in the same individuals. The model must approximate the known changes in peripheral IGF-1 and IL-6 levels with aging, as well as their relationship to mobility performance, disability, and death, and we had to use data from multiple published studies to fit parameters in the model.

As the relationship between IGF-1 and IL-6 does not depend on prevalence score of mobility and disability, we can identify parameters governing the dynamics of these biomarkers independent of other components. In the Cappola study, IL-6 ranged between 0.4 and 10.1 pg/ml, however many cases of much higher levels of IL-6 (up to 43.5 pg/ml) have been observed (Said et al., 2021). Thus, we set the maximal allowable value of IL-6 to be 25 pg/ml which we use in the model. Similarly, in Cappola study the maximal observed value of IGF-1 was 300 ng/ml. Other clinical studies reported similar values: 15–315 ng/mL for Czech population of 75–80 year old (Kucera et al., 2015), 50–180 ng/mL for Brazilian population of the same age (Rosario, 2010) and 45–305 ng/ml for Chinese population (Zhu et al., 2017). Informed by these studies, we set the maximal value of IGF-1 to be 400 ng/ml. This experimental data allowed us to estimate values of *kp*_*IGF1*,_ *kd*_*IGF1*,_ *kp*_*IL6*_ and *kd*_*IL6*_ parameters.

To estimate values of the parameters ks_IGF1_ and ks_IL6_ we fitted these two parameters to the 5 years time courses of IGF-1 and IL-6 blood serum levels. We start from levels reported in Cappola study and estimate annual changes in biomarker levels using the data from multiple clinical sources. At the beginning of the Cappola study, the average age of the cohort is 77.6 years and the mean IL-6 serum level is 3.14 pg/ml. We adopt the changes in IL-6 level reported by Said et al., 2020 (Said et al., 2021), which finds a linear increase in IL-6 of 0.05 pg/ml per year in healthy adults. Those data are roughly consistent with other data we could find. Kiecolt-Glaser et al. (2003) reported an average annual increase of IL-6 levels in two cohorts of people between 55 and 95 years old. For a cohort of caregiver adults that are under constant stress, the average annual increase was 0.063–0.237 pg/ml/yr from ages 77.6–82.6 years old. For healthy adults the annual change was smaller (less than 0.071) pg/ml/yr) but this result was not significant. Albani et al. (2009) reported an average annual increase in IL-6 of 0.0178 pg/ml/yr between the ages of 77.6 and 82.6 for an elderly Italian population.

At the beginning of the Cappola study, the mean IGF-1 value was 107.8 mcg/L. Kucera et al. (2015) reported an annual decrease of IGF-1 serum levels to be 1.98 ng/ml per year for women. Rosario (Rosario, 2010) reported an average annual decrease of 1.4 ng/mL for a younger population of 45–70 years old. Additionally, Vestergaard et al. (2014) found a decrease in total serum IGF-1 in healthy adults of 1.48 percent per year. Thus, we set the annual decrease of IGF-1 levels in our model to be 1.95 ng/mL per year.

To estimate the prevalence score of mobility, disability and mortality, we use data from Cappola et al., 2003. They provide data for 758 individuals subdivided into four cohorts (quartiles) of IL-6/IGF-1 combinations (398 patients with high IGF-1/low IL-6 levels at the beginning of observations, 128 patients with high IGF-1/high IL-6 levels, 142 patients with low IGF-1/low IL-6 levels, and 50 patients with low IGF-1/high IL-6 levels) over a period of 60 months for a target group aged 60–65 years old at baseline. The cut-offs in IGF-1 and IL-6 levels were selected by Cappola et al. based on their prior studies suggesting a threshold effect for mobility tasks at approximately these levels. The data available for each cohort includes the number of individuals having mobility disability at the beginning of observations, the number of individuals having mobility disability at 36 months, and timecourse survival data over 60 months. Resulting gaps were inferred based on linear approximation.

Finally, the contribution of mobility disability to mortality rates is defined by the parameter k_extra_ (effectively an amplifier of the mortality rate of the mobile population resulting in increased death prevalence score in the mobility disability population). To estimate this parameter, we looked into studies of mortality rates. Gilmour and Ramage-Morin (Gilmour and Ramage-Morin, 2021) studied mortality over a 5 year period among 29,302 Canadians. They found that older adults who were frail were 3.5 times more likely to die than those who were not frail. In the Cappola et al. (2003) study, mortality over 5 years in the least frail population (the 4^th^ cohort, high IGF-1 and low IL-6 levels) was 2.5 times less than in the first cohort (low IGF-1 and high IL-6 levels) with the most vulnerable population. Hao et al. (2018) found that in a Chinese population admitted to the geriatric ward of a hospital, frail patients were 2.09–2.18 times more likely to die within 3 years than non-frail patients. Based on these sources, we choose the value of k_extra_ to be 2.5. Having estimated the value of k_extra_ from the literature, we identify the values of k_loss_, k_gain,_ k_mort_ and k_longevity_ by fitting the system of differential equations for M(t) and MD(t) to the values of MD and M from Cappola study.

Additional details about the source data and the fitting procedures are provided in the Supplementary Material.S1

### 2.3 Model simulations

After unknown parameters for each cohort were identified using parameter estimation, the system of differential equations can be solved numerically. This produces time-course simulations of the model that predict prevalence score of mobility and mobility disability for humans with different initial serum blood levels of IGF-1 and IL-6. We used VCell (Moraru et al., 2008) for such simulations, and emphasis was placed on modeling trends since exact values were often not available. Therefore, our goal was to predict general trends from a qualitative, and, to a lesser extent, quantitative perspective. The model and simulation results are available in VCell database that is publicly accessible from within VCell software (http://vcell.org/). While no registration is required, one must download and install the software to access the model and check all simulation results. The model is available by searching for Aging_Phenotypes in BioModels.

## 3 Results

In Section 3.1 we demonstrate that the molecular mechanism described in the Methods section leads to bistability on the molecular level, where for each pair of initial levels of IGF-1 and IL-6, values converge to two stable values. In Section 3.2 we demonstrate that this bistability is carried through into the phenotypic model. In Section 3.3 we describe these two stable clinical outcomes. Finally, in Section 3.4 we identify regions of initial IGF-1 and IL-6 levels that correspond to these two distinct outcomes. Specifically, we identify a set of pairs of IGF-1 and IL-6 initial values that define a curve where all combinations of initial values from two sides of the curve lead to different phenotypical outcomes.

### 3.1 Emergence of bistability in IGF-1 and IL-6 levels

**FIGURE 3:**
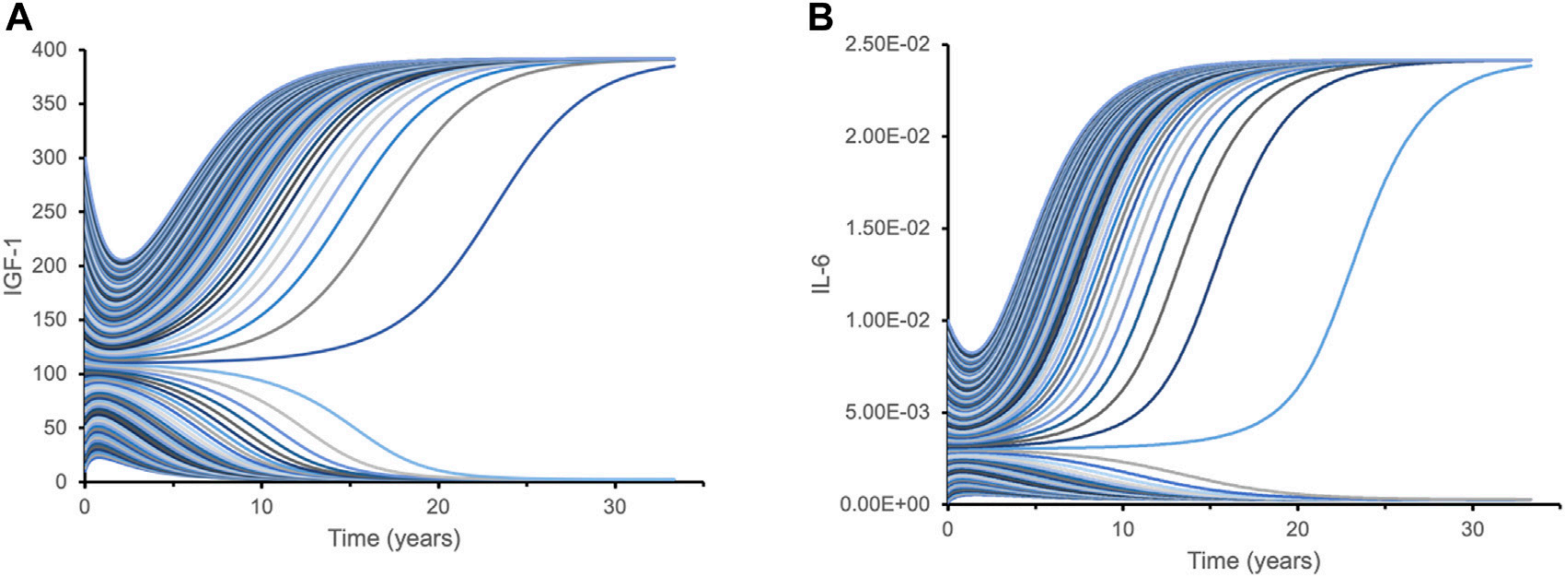
Dynamic time course simulations of biomarkers demonstrate bistability with respect to their initial levels. Each line represents a time course of IGF-1 **(A)** and IL-6 **(B)** for different initial combinations of IGF-1 and IL-6 biomarkers. Variations in initial levels (time point 0) lead to just two qualitatively distinct outcomes over extended time periods: either close to the physiological maximum, or close to physiological minimum. Such bistability is caused by the mutual inhibition mechanism for IGF-1 and IL-6 pathways. This hypothetical model is designed to illustrate isolated interactions between IGF-1 and IL-6 based on their initial levels since other effects and factors are not considered.

After estimating model parameters as described above, we performed multiple simulations using different initial conditions corresponding to the known variance in IGF-1 and IL-6 levels of the studied cohorts: initial IGF-1 concentrations ranged from 40 mcg/L to 300 mcg/L, while initial IL-6 concentrations varied from 0.001 mcg/L to 0.01 mcg/L. The results showed that the inverse correlation between IGF-1 and IL-6 (akin to “mutual inhibition”) leads to two distinct outcomes (steady states) over time (Figure 3). Depending on the initial values of one biomarker, the values of another biomarker over extended time periods converge towards either close to the physiological maximum or close to the physiological minimum—two qualitatively very different outcomes.

### 3.2 Emergence of switch-like behavior in clinical outcomes

**FIGURE 4:**
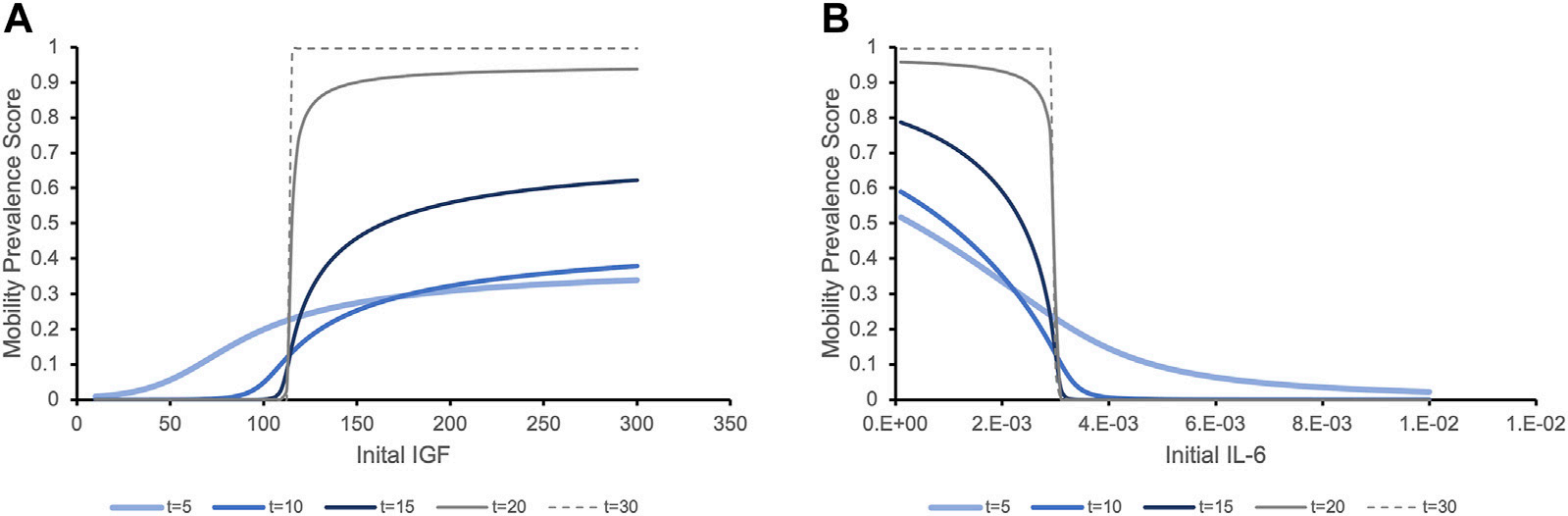
The prevalence score of being mobile depending on the initial levels of serum biomarker IGF-1 **(A)** and IL-6 **(B)** exhibit switch-like behavior with small changes in initial levels around critical values leading to dramatically different outcomes at later times. **(A)**. The prevalence score of mobility are simulated for an individual with the population mean level of IL-6 and varying initial levels of IGF-1. The prevalence score of being mobile at 5 years after initial measurement of IGF-1 gradually increase as the initial IGF-1 level increases. As time increases, the critical threshold between negative and positive outcomes becomes more evident. Although very few people survive to 20 or 30 years, the prevalence score of these surviving people to be mobile is significant if they had initial IGF-1 values above the threshold, while for people with IGF-1 levels below this value, the prevalence score of mobility is almost zero. **(B)**. Similar trends in the prevalence score of mobility is expressed when we consider a population with the same initial level of IGF-1 and varying levels of IL-6. With initial IL-6 levels above a critical threshold, the prevalence score of being mobile rapidly decline. Below the threshold, the situation is the opposite.

We then tested our hypothesis that this IGF-1/IL-6 relationship leads to switch-like behavior in the predicted clinical outcomes. For different initial values of one biomarker, we observed two different trends in the prevalence score of mobility. Figure 4A illustrates that for individuals with initial IGF-1 below a critical threshold (vertical line), the prevalence score of mobility decreases over time. When the initial IGF-1 value is above the threshold, the clinical outcomes are the opposite: the prevalence score of mobility increases over time. This effect significantly increases over time. While at 5 years individuals with the same initial levels of IL-6 but distinct IGF-1 levels have the prevalence score of mobility roughly proportional to the initial IGF-1 levels, at longer time periods (20 and 30 years) we see a striking effect of the different initial levels of IGF-1: even small variations around the critical value exert a profound effect on the mobility outcome, a switch-like behavior. Conversely, Figure 4B demonstrates a similar pattern in the predicted future of mobility outcome for individuals with the same initial level of IGF-1 but with different initial levels of IL-6. Note that while biomarker levels express true bistability (over a period of time longer that the lifespan of individuals in the study), clinical outcomes never reach a true steady state because of to-death transition, so they express “switch-like” and not bistable behavior.

### 3.3 Emergence of two drastically different clinical phenotypes

**FIGURE 5:**
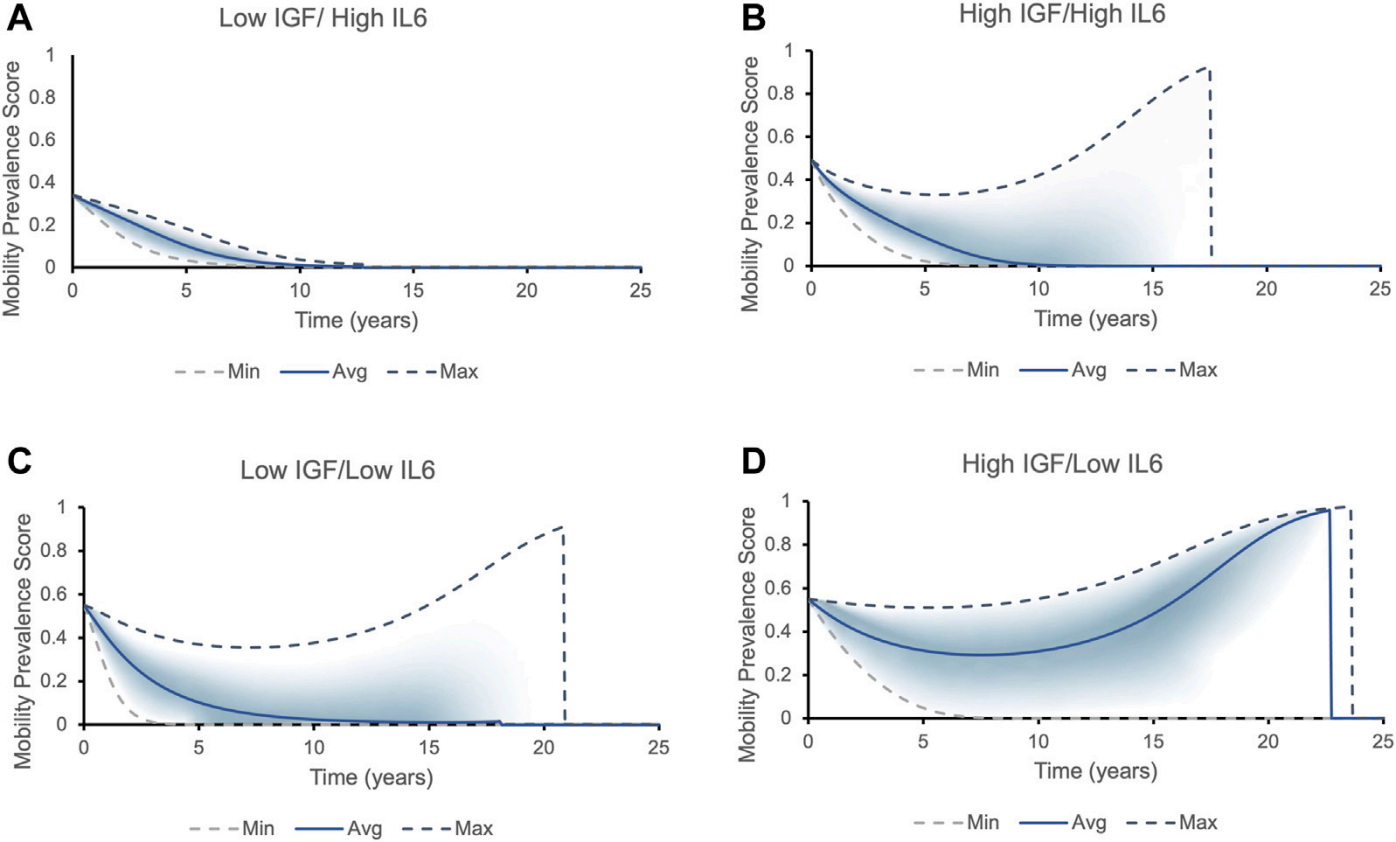
The mobility prevalence score over time for individuals with different initial levels of IGF1 and IL6. Top and bottom lines correspond to the best (highest IGF-1 and lowest IL-6 levels) and worst (lowest IGF-1 and highest IL-6 levels) phenotypic outcomes possible within that each cohort, respectively. The solid line corresponds to the average values of IGF-1 and IL-6 within each cohort. The prevalence score of remaining mobile for each cohort falls into the area between the two dashed lines, with most individuals falling into the shaded area. Two possible outcomes present themselves in all cohorts except for Low IGF/High IL6: either the longer an individual lives, the higher is the prevalence score of remain and die being non-frail/mobile (the prevalence score increases to one and then suddenly drops to zero, indicating a death of an individual - mobile phenotype), or the chances of being mobile rapidly decrease (frail phenotype/declines in mobility). **(A)** All individuals with initial levels of low IGF-1 and high IL-6 belong to the frailty disability phenotype: the prevalence score of being mobile decreases rapidly. **(B)** Individuals with initial levels of high IGF-1 and high IL-6 can fall into both phenotypes. The individual’s mobility prevalence score decreases initially, but then start increasing: the longer person lives, the higher their chances to remain and die being mobile. The average individual has roughly 30% chances of dying in a mobile state. **(C)** Majority of individuals with initial levels of low IGF-1 and low IL-6 express frailty phenotype. **(D)** Majority of individuals with initial levels of high IGF-1 and low IL-6 case belong to non-frail phenotype.

We further examined the influence of the switch-like effect of initial biomarker on mobility outcome. We performed simulations representing a hypothetical individual with mean biomarker values of the four distinct cohorts studied. Figure 5 demonstrates that different combinations of high/low values for IGF-1 and IL-6) (Cappola et al., 2003) produce different mobility prevalence score within each quartile.

**FIGURE 6:**
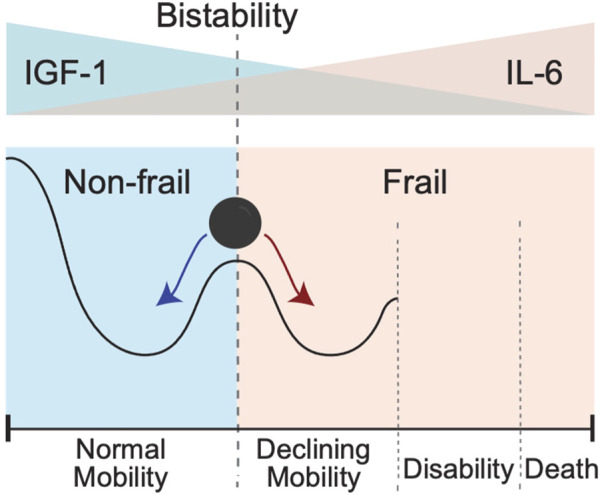
Bistability of IGF-1 or IL-6 blood levels could create conditions for the bistability involving two different clinical states pertaining to absence or presence of a frailty phenotype. In this schematic cartoon in the top panel we show the initial levels of IGF-1 and Il-6: from high IFG-1 (blue)/low IL-6 (pink) on the left to the opposite low IFG-1 (blue)/high IL-6 (pink) on the right. In the bottom panel we show the corresponding resulting trends in mobility. Going from left to right, the black line shows a sketch of trajectory an individual experiences as IGF-1 level goes down and IL-6 level goes up. The exact form of trajectory depends on multiple factors that are not considered in this simplified cartoon, but individuals with IGF-1 above a certain critical value for IGF-1 and/or those with IL-6 values below a certain critical value for IL-6 will demonstrate the presence of the normal mobility phenotype. In contrast, presence of lower IGF-1 and/or higher IL-6 levels will favor declines in mobility performance, leading to disability and an ultimate death. Individuals with initial levels of IGF-1 and IL-6 around the critical threshold (schematically shown as a vertical dashed line) are pushed toward either normal mobility or declining mobility.

Results of modeling presented in Figure 5 suggest that initial IL-6 and IGF-1 levels may help to stratify individuals into two contrasting phenotypes (Figure 6). Individuals in the “high-mobility” group have IGF-1 levels which do not decrease (and may in fact increase over time), and have increased prevalence score of surviving in a mobile state. In contrast, individuals in the “low-mobility” group have decreasing levels of IGF-1 and decreasing prevalence score of mobility through their remaining lifetime.

### 3.4 Initial level of biomarkers as phenotype predictors

**FIGURE 7:**
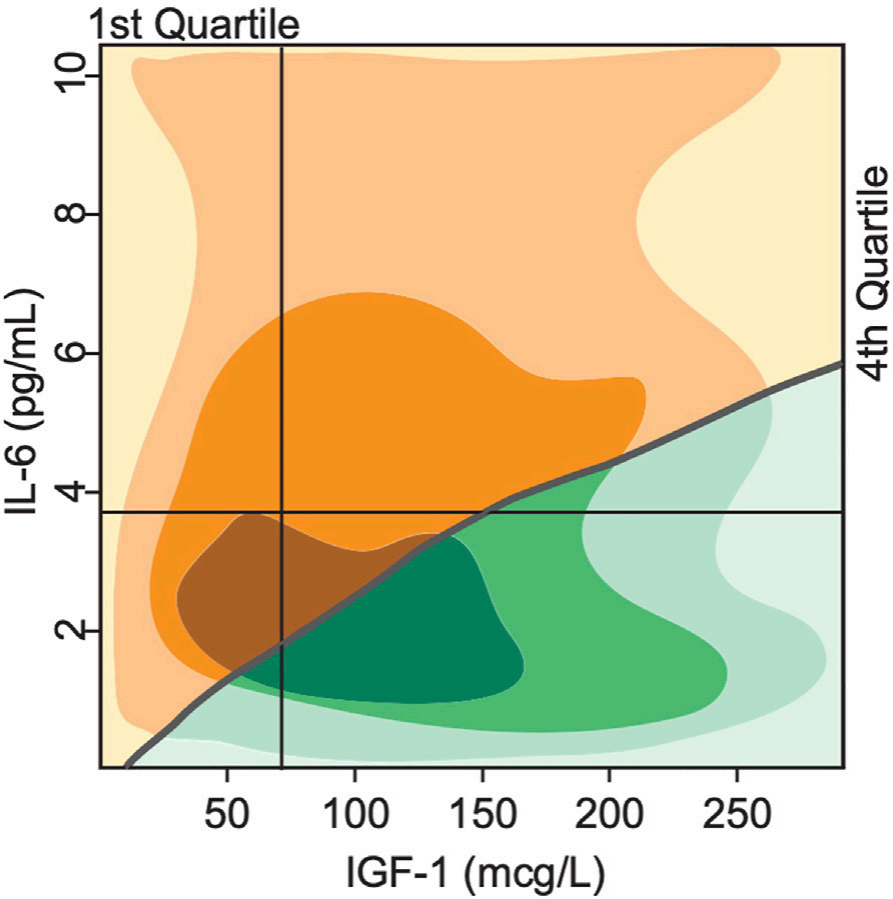
Bistability in biomarkers defines two distinct phenotypes based on deterministically computed mobility disability outcomes. Superimposing the switch points produced by the mathematical model over the experimental study population of Cappola et al., 2003 illustrates a critical threshold (grey line) that divides the population into 2 groups. In the first group (green), the steady state serum level of IGF-1 is high, while IL-6 levels are low, indicating more positive phenotypic outcomes such as lower prevalence score of frailty and declines in mobility performance throughout the lifespan of the group. In the second group (yellow), the steady state serum level of IGF-1 is low, while IL-6 is high, indicating more negative phenotypic outcomes such as higher prevalence score of frailty and declines in mobility performance throughout the lifespan of the group. The shading indicates areas with majority of study population (the darker—the more subjects).

The exact trajectory of declining mobility and disability and death is subject to individual variability and happenstance and impossible to derive from just two biomarkers. Nevertheless, one can estimate the strength of the predictive value of these biomarkers. We performed a phase plane analysis of the two distinct clinical states or phenotypes discussed above in relation to the initial levels of IGF-1 and IL-6. Figure 7 shows the regions corresponding to either mobile/resilient (green) or less mobile/frail/disabled (orange/yellow) phenotypes in the overall population according to our simulations. These regions are superimposed on the plot of Cappola et al. (Cappola et al., 2003) that stratified the study population into 4 cohorts according to the IGF-I and IL-6 serum levels. The upper left quadrant identifies participants in the highest risk group (illustrated in Figure 5A), and the lower right quadrant shows those in the lowest risk group (illustrated in Figure 5D).

Our simulation results classify all individuals in the 1^st^ quadrant as having the frail phenotype, while individuals in the 2^nd^, 3^rd^ and 4^th^ quartiles will have either the resilient of frail phenotype according to the position relative to the curve. These results show a much more fine-grained predictive power of our model compared to the results of Cappola et al.

## 4 Discussion

### 4.1 Mathematical modeling as a tool to predict dynamic behavior

The classical approach to the analysis of the effects of biomarkers on clinical phenotypes is statistical modeling: a set of statistical assumptions on a generation of data that then allows the inference of the probability of certain events or relationships between variables. In contrast, dynamical models are based on a set of assumptions on mechanisms of interactions. Dynamical models can go beyond the predictions of statistical models, but at the cost of making assumptions that may not be explicitly validated by existing data. We have built a dynamical model that predicts functional outcomes over time based on hypothesized mechanisms of interactions between biomarkers IGF-1 and IL-6 and their relationship to mobility and frailty phenotypes. In the absence of knowledge about molecular mechanisms of pathway interactions, the dynamical model serves as proof of concept. However, as more data on molecular mechanisms and their relationship to clinical outcomes become available, dynamical modeling will be able to provide more precise predictions, while also helping to move the field from a study of association to approaches guided by mechanisms. However, it is important to emphasize that even if detailed molecular mechanisms are not known, dynamical models can be constructed using phenomenological approximations of high-level hypotheses (“black box” approach) that yield valuable insights and have significant predictive power, as shown in this study.

One of the challenges in capturing the full complexity of human aging through mathematical models stems from the fact that the rate at which individuals age and become disabled demonstrates great heterogeneity both between subjects, as well as over time within the same individuals (Guralnik et al., 1996). Clinical and research evidence indicates that specific states of increased vulnerability or frailty can be identified in subsets of older adults using criteria for the existence of distinct clinical presentations or frailty phenotypes (Fried et al., 2001; Bouchonville and Villareal, 2013). Our mathematical model focuses on IL-6 and IGF-1, a pair of biomarkers with opposite effects on the likelihood of developing mobility disability (Cappola et al., 2003). The model suggests the existence of critical IGF-1 and IL-6 blood levels where even small variations around these values may lead to strikingly different outcomes in mobility performance, a feature that plays a central role in the development of frailty and functional dependence. This demonstrates a switch-like behavior in predicting mobility disability: slight differences in the initial level of either biomarker do not show significant differences in the short term, but over the long term (10–20 years) result in completely distinct overall risks of declining mobility performance. This suggests that the synergistic but inverse relationship between IGF-1 and Il-6 may facilitate a “switch” from a state in which a resilient individual has a low risk of future disability towards another phenotypically-distinct state or phenotype which is associated with frailty and a high risk of future disability (Figure 4)—a hallmark of bistability.

Bistability represents a fundamental property of certain systems which can exist in either one of two stable states (Tyson et al., 2003). Such systems function in a manner similar to a toggle switch, transitioning from one stable state to another (Tyson et al., 2003; Wilhelm, 2009) once the parameters controlling the system’s function start diverging in one direction or another around certain critical values. Within biological systems, bistability is key for understanding decision-making processes such as cell cycle progression, cell death, cellular differentiation, and development (Tyson et al., 2003; Wilhelm, 2009). In our manuscript, a double-negative feedback loop leads to bistability; however, both positive and double-negative feedback loops can also generate bistability, resulting in the emergence of switches leading to all-or-nothing decisions involving oocyte maturation, calcium signal transduction, and cellular polarity (Ferrell, 2002; Brandman and Meyer, 2008). The principle used to construct our “proof-of the-concept” model can be applied to many different biological systems where mutually antagonistic pathways are assumed to be involved in the development of contrasting phenotypes.

### 4.2 Potential clinical insights from our model

Our findings provide mathematical evidence in support of the concept that interactions among biomarkers and associated pathways may show a certain type of synergy that can ultimately lead to bistable behavior. These observations do not diminish the key importance of stochastic or random events within the complex and variable multifactorial pathways which lead to disease, disability, or death. Rather, these considerations provide potential insights in support of additional layers of systemic organization and complexity that may help define those clinical conditions or phenotypes which clinicians and investigators have observed within subsets of older adults. These could include different categories of specific frailty states such as the Fried Frailty Phenotype which manifests as the combination of weight loss, exhaustion, weakness, slow walking speed and low physical activity (Fried et al., 2001), or obesity-related frailty with muscle weakness and poor physical performance (Baumgartner et al., 2004). Another common clinical scenario in geriatrics practice includes acute changes in muscle weakness or mental status that frequently lead to emergency room visits and imaging studies, without any positive finding for stroke, transient ischemic attack, urinary tract infection, or other metabolic abnormalities. Explanations for such events usually are described as depletion of reserve (physical or cognitive) in each individual to the threshold level of showing clinically evident symptoms (and thus acute changes become apparent). However, the mechanism of bistability and switch-like behavior described in this paper may represent an alternate or complementary explanation of such clinical observations.

Point of care testing represents the capacity to perform a diagnosis using laboratory tests obtained in the course of a clinical visit, with such results helping to guide the rapid formulation of an individualized treatment plan (Kost, 2002). Based on historical precedents, it remains unlikely that biomarker data obtained from large longitudinal studies of aging will be rapidly translated into permitting or facilitating decision-making at the bedside. Nevertheless, principles uncovered in our study could help advance the progress of aging research in several important directions. Above all, our finding that the co-existence of mutually-inhibitory biomarkers for disability may lead to the emergence of bistability supports the existence of specific frailty states or phenotypes that develop late in life. Such considerations may help provide an added justification and a clearer focus on specific targets for future pathophysiologic studies into physical, cognitive or mental frailty (examples include, among others, sundowning or acute confusion towards late afternoon which often resolves towards evening or episodic psychotic events) and other geriatric syndromes. Finally, it will be especially important to extend such work towards the identification of specific mechanisms through which mutually inhibitory pathways interact with each other and contribute to bistability. Such more detailed knowledge combined with correspondingly refined dynamical modeling will likely offer particularly important novel targets for the development of innovative future therapies (Inouye et al., 2007).

## Data Availability

The original contributions presented in the study are included in the article/Supplementary Material, further inquiries can be directed to the corresponding authors.
