# Supplementary material for: Dynamic model assuming mutually inhibitory biomarkers of frailty suggests bistability with contrasting mobility phenotypes
Source: Front Netw Physiol. 2023 May 4;3:1079070. doi: 10.3389/fnetp.2023.1079070 (PMC10192762; doi:10.3389/fnetp.2023.1079070)
Supplement: Supplementary file 1 [file DataSheet1.docx]

**Supplemental material**

**Parameter fitting with VCell:** The parameter fitting was performed given the lack of exact values of molecular interactions among IGF-1 and IL-6 pathways. We assumed that IGF-1 and IL-6 pathways inhibit each other, and each factor is produced and degraded at the constant rate not changing over time. We have to estimate the values of 6 parameters: two production rates kp_IGF1_ and kp_IL6_, two degradation rates kd_IGF1_ and kd_IL6_ , and two parameters ks_IGF1_ and ks_IL6_ that define the strength of inhibition of the IGF1 and IL6 production by the opposite pathway.

The model is under-defined, as we have more kinetic parameters than distinct data sets to allow for proper fitting. Thus, we start by fitting production and degradation rates, so that their ratio for each risk factor defines the steady state value consistent with experimental data. In the Cappola study IL-6 ranged between 0.4 and 10.1 pg/ml, however many cases of much higher levels of IL-6 (up to 43.5 pg/ml) have been observed (Said et al., 2020). Thus, we set the maximal allowable value of IL-6 to be 25 pg/ml that we use in the model. Similarly, in Cappola study the maximal observed value of IGF-1 was 300 ng/ml. Other clinical studies reported similar values: 15-315 ng/mL for Czech population of 75-80 year old (Kucera et al., 2015), 50-180 ng/mL for Brazilian population of the same age (Rosario et al., 2010) and 45-305 ng/ml for Chinese population (Zhu et al., 2017). So, we set the maximal value of IGF-1 to be 400 ng/ml that we use in the model.

To approach these maximal values, we compute the ratio of kp_IGF1_ to kd_IGF1_ to be 400 s^-1^, the ratio of kp_IL6_ to kd_IL6_ to be 0.025 s^-1^ to give maximum asymptotic value of IL-6 and IGF-1 that is around the upper end of the physiologically possible ranges.

To estimate values of the parameters ks_IGF1_ and ks_IL6_ we used VCell parameter estimation (Aging_Phenotypes model by user aging_project, Molecular_Mechanism application) fitting those two parameters to the clinically observed timecourses of IGF-1 and IL-6 blood serum levels over 5 years. Those timecourses will be discussed below. Note that the initial values of ks_IGF1_ and ks_IL6_ were chosen so that the strengths of IGF-1 and IL-6 inhibitions are on the same scale (given that scales of IGF-1 and IL-6 values are 6 orders of magnitude different).

**Estimates of IL-6 levels over time:**  We used data from multiple clinical sources. At the beginning of the Cappola study, the average age of the cohort is 77.6 yrs and the mean IL-6 serum level is 3.14 pg/ml. We adopt the changes in IL-6 level reported by Said et al., 2020, that finds a linear increase in IL-6 of 0.05 pg/ml per year in healthy adults. Those data are roughly consistent with other data we could find. Kiecolt-Glaser et al., 2003 reported average annual increase of IL-6 levels in two cohorts of people between 55 and 95 years old. For a cohort of caregiver adults that are under constant stress, the average annual increase was 0.063-0.237 pg/ml/yr from ages 77.6 to 82.6 years old. For healthy adults the annual change was smaller (less than 0.071) pg/ml/yr) but the confidence interval didn’t allow to use these data. Albani et al. (2009) reported an average annual increase in IL-6 of 0.0178 pg/ml/yr between the ages of 77.6 and 82.6 for an elderly Italian population.

We use ng/mL to be consistent with units of IGF-1, so we scale reference levels by 10-4 to convert into the same units.

| T (months) | Reference levels (pg/mL) | Model IL-6 levels (ng/mL) |
| --- | --- | --- |
| 0 | 3.14 (Cappola et a., 2003) | 0.00314 |
| 12 | 3.19 (Said et al., 2020 | 0.00319 |
| 36 | 3.29 Said et al., 2020 | 0.00329 |
| 60 | 3.39 Said et al., 2020 | 0.00339 |

Table 1. Estimated levels of Il-6 in the genera study population at 12, 36 and 60 months.

**Estimates of IGF-1 levels over time:** At the beginning of the study by Cappola study the average age of the cohort is 77.6 yrs and the mean IGF-1 value is 107.8 mcg/L. Kucera et al. (2015) reported an annual decrease of IGF-1 serum levels to be 1.98 ng/ml per year for woman of Rosario (2009) reported an average decrease of 1.4 ng/mL for younger population of 45-70 years old. Additionally, Vestergaar et al. (2014) found a decrease in total serum IGF-1 in healthy adults of 1.48 percent per year. Thus, we set the annual decrease in our model to be 1.95 ng/mL per year.

| T (months) | Reference level (ng/ml) (Same) |
| --- | --- |
| 0 | 107.8 (Cappola et a., 2003) |
| 12 | 105.85 (Kucera et al., 2015) |
| 36 | 101.95 (Kucera et al., 2015) |
| 60 | 98.05 (Kucera et al., 2015) |

Table 2. Estimated levels of IGF-1 in the general study population at 12, 36 and 60 months.

**Defining parameters for changes in mobility**. The fitted values of the 5 parameters describing molecular mechanism are used in the clinical module that describes the effect of IGF-1 and IL-6 blood serum levels on changes in mobility of individuals. The clinical module is characterized by 5 parameters: k_loss_ and k_gain_ defining the loss and gain of mobility, k_mort_ is the mortality rate constant for the mobile subpopulation of study, k_extra_ defines an increased death probability for people with mobility disability compared to mobile population, and k_longevity_ defines the exponential increase in the probability of death with age, effectively setting the maximal survival of the studied population. We assume that k_death_ is the same for all quadrants (fitted in Application “Average phenotype” to the average values of mobility and survival across all cohorts) and all changes among people in different cohorts are driven by k_m_ is the mortality rate constant for the mobile subpopulation of study, k_extra_

We are making a simplifying assumption that odds of mobility disability, mobility and death are affected only by biomarker levels and not directly by age. However, there is an implicit assumption based on Said 2020 and Kucera 2015, that age is correlated with biomarker levels. This assumption allows us to still account to some degree for deviations from the mean age of the study population when interpreting the model as a diagnostic tool for an individual with certain combinations of IGF-1 and IL-6. To improve the model’s capabilities as a diagnostic tool, more time course data for IGF-1, IL-6, and mortality is needed to allow for fitting for each specific age.

**Estimating the contribution of mobility disability to the mortality**

The contribution of mobility disability to the mortality is defined by the parameter k_extra_ that is an amplifier of the mortality rate for mobile population to obtain increased death odds in mobility disability. To estimate this parameter, we looked into studies of mortality rates. Gilmour and Pamela L. Ramage-Morin (2021) studied mortality over the five years period among 29,302 Canadians. They found that older adults who were frail were 3.5 times more likely to die as those who were not frail. In Cappola et al. (2003) study the mortality over 5 years in the least frail population (the 4^th^ cohort with the high IGF-1 and low IL-6 levels) was 2.5 time less that in the first cohort with the most vulnerable population. Hao et al (2018) found that in a Chinese population admitted to the geriatric ward of a hospital, frail patients were 2.09-2.18 times more likely to die within three years as non-frail patients. Based on these sources, we choose the value of k_extra_ to be 2.5 in correspondence with Cappola data and accounting that it is still within ranges described in other publications.

**Estimating Mortality and Mobility Disability for different cohorts**

To find the values the values of the remaining 4 parameters describing the changes in clinical outcomes (k_loss_ and k_gain_ defining the loss and gain of mobility, k_mort_ is the mortality rate constant for the mobile subpopulation of study, and k_longevity_  describing the exponential increase in the probability of death with age of the general population) we combined the data about molecular changes we discussed above with the clinical outcomes from Cappola et al. (2003) presented for each cohort independently.

At the beginning of the study by Cappola study the average age of the cohort is 77.6 yrs and the number of people in the total population of 718. We assume that study population is homogeneous and the average age is increasing^1^. Figure 3 of the Cappola study provides the percentage of people surviving in each quadrant over the course of five years after the start of the study. We used this plot to find the number of people deceased after 12, 36, and 60 months for each quadrant.

Cappola study provides the initial number of participants with mobility disability, as well as the percentage of the remaining members of each cohort with mobility disability after 36 months. Since Cappola data of mortality extend to 5 years’ timeframe, we wanted to extrapolate the mobility disability numbers to the same timeframe to get more data points for fitting.

Liao and Chang (2020), showed that odds of disabilities can follow a sigmoidal curve, which is corroborated visually by figure 1A from Leveille et al (2000). Thus, we fit each cohort using a sigmoidal function y (the percentage of the remaining population with mobility disability) depending on time t in the form $y=d+\frac{a-d}{1+\left( \frac{t}{c} \right)^{b}}$. Here *d* is the upper asymptote (the final percentage of disabled people, which we set to be 100%), *a* is the lower asymptote (the starting percentage of mobilitydisabled people in the cohort). The value of *a* was found from a 2000 study by Leveille et al that identified the average mobilty disability in the population of age 65 years old to be 18 percent. Note that it is the youngest age in the Cappola study. We assume that all four cohorts have the same lower asymptotic value for mobility disability. This assumption is based on the inherent idea of our model that subtle differences in IGF and IL6 are exacerbated over time, therefore at younger ages the differences between the cohorts should be less dramatic. Additionally, Ferrucci et al (2016), found that the critical age for mobility decline in the general population is at about 70 years whereas the average initial age of the Cappola study population is 77.6 years. This also points to the idea that at age 65, there should be lesser differences in mobility disability between the cohorts.

Values of *c* (the inflection point) and *b* (the slope factor) are fitted based on values of mobility disability at the initial time (Table 1 of Cappola study) and at 3 years observation (Table 3 of Cappola study). We set up a system of equations to solve for the slope factor and inflection point. The fitted values (Table 3) were used to find estimated values of mobility disability at 12 and 60 months.

| Parameter | High IGF-1, Low IL-6 | Low IGF-1, Low IL-6 | High IGF-1, High IL-6 | Low IGF-1, High IL-6 |
| --- | --- | --- | --- | --- |
| *b* | 4.0435 | 8.6288 | 6.3093 | 8.6632 |
| *c* | 15.0445 | 13.68295 | 13.4363 | 13.4751 |

Table 3. Fitted parameter values for a sigmoidal curve defining mobility disability in the four cohorts.

| Cohort with the initial high IGF-1 and low IL-6 levels | Initial assessment | 12 months | 36 months | 60 months |
| --- | --- | --- | --- | --- |
| Deceased people | 0 | 7 | 36 | 92 |
| People with mobility disability | 179 | 198 | 224 | 219 |
| Mobile people | 219 | 193 | 138 | 87 |
| Total | 398 | 398 | 398 | 398 |

Table 4. Changes in the number of people in the first cohort (average level of IGF-1 is 148 ng/L and the average level of IL-6 is 0.00206 ng/mL) having mobility disability

| Cohort with the initial low IGF-1 and low IL-6 levels | Initial assessment | 12 months | 36 months | 60 months |
| --- | --- | --- | --- | --- |
| Deceased people | 0 | 4 | 17 | 37 |
| People with mobility disability | 64 | 80 | 100 | 96 |
| Mobile people | 78 | 58 | 25 | 9 |
| Total | 142 | 142 | 142 | 142 |

Table 5. Changes in the number of people in the second cohort (average level of IGF-1 is 69 ng/L and the average level of IL-6 is 0.00206 ng/mL)having mobility disability

| Cohort with the initial high IGF-1 and high IL-6 levels | Initial assessment | 12 months | 36 months | 60 months |
| --- | --- | --- | --- | --- |
| Deceased people | 0 | 10 | 36 | 45 |
| People with mobility disability | 65 | 72 | 71 | 73 |
| Mobile people | 63 | 46 | 21 | 10 |
| Total | 128 | 128 | 128 | 128 |

Table 6. Changes in the number of people in the third cohort (average level of IGF-1 is 148 ng/L and the average level of IL-6 is 0.00623 ng/mL) having mobility disability

| Cohort with the initial low IGF-1 and high IL-6 levels | Initial assessment | 12 months | 36 months | 60 months |
| --- | --- | --- | --- | --- |
| Deceased people | 0 | 5 | 13 | 23 |
| People with mobility disability | 33 | 32 | 30 | 24 |
| Mobile people | 17 | 13 | 7 | 3 |
| Total | 50 | 50 | 50 | 50 |

Table 7. Changes in the number of people in the fourth cohort (average level of IGF-1 is 69 ng/L and the average level of IL-6 is 0.00623 ng/mL) having mobility disability

We used VCell parameter estimation (aging_study model by user aging_project, cohort 1-4 applications) fitting those four parameters to the timecourses over 5 years described below. After fitting, we have obtained the following values of the parameters:

| Parameter | Average biomarker values | High IGF-1, Low IL-6 | Low IGF-1, Low IL-6 | High IGF-1, High IL-6 | Low IGF-1, High IL-6 |
| --- | --- | --- | --- | --- | --- |
| k_loss_ | 5.528 | 7.183 | 10.555 | 4.622 | 2.607 |
| k_gain_ | 1e-8 | 1e-8 | 1e-8 | 1e-8 | 1e-8 |
| k_mort_ | 1.720e-3 | 1.364e-3 | 1.470e-3 | 2.547e-3 | 3.049e-3 |
| K_longevity_ | 1.372e-2 | 1.372e-2 | 1.372e-2 | 1.372e-2 | 1.372e-2 |
| Maximal lifespan using floor from best case scenario (months) | 228 | 284 | 259 | 223 | 187 |

Table 8. The values of parameters describing mortality and mobility disability changes in each cohort for fixed K_longevity_

We did systematic fitting of those parameters and concurrent simulation of phenotypes with different initial values of IGF-1 and IL-6 and different data on mobility/disability and mortality. The qualitative results of the manuscript do not change.
